# Superbase ionic liquid mediated solubilization of curcumin for improved bioavailability and anticancer efficacy

**DOI:** 10.1038/s41598-026-44082-7

**Published:** 2026-04-17

**Authors:** Meena Bisht, Maria C. Gomes, Filipe Hobi Bordon Sosa, João F. Mano, Siddharth Pandey, Sónia P. M. Ventura, João A. P. Coutinho

**Affiliations:** 1 Department of Chemistry, Sri Venkateswara College, University of Delhi, Dhaula Kuan, New Delhi, 110021 India; 2 Department of Chemistry, CICECO-Aveiro Institute of Materials, University of Aveiro, 3810-193 Aveiro, Portugal; 3 Department of Chemistry, Indian Institute of Technology Delhi, Hauz Khas, New Delhi, 110016 India

**Keywords:** Curcumin, Superbase ionic liquid, Solubility, Anticancer activity, Biochemistry, Biotechnology, Cancer, Chemistry, Drug discovery

## Abstract

Curcumin is a promising natural drug for the treatment of various diseases ranging from cancer to Alzheimer’s. However, a major hindrance in its use as a drug is its low aqueous solubility, rapid degradation, and poor cellular uptake. In recent years, the use of ionic liquids (ILs) in biomedical applications has gained significant attention due to their unique properties and tunability. In this study, we demonstrate the capacity of the superbase ionic liquid (SBIL) 5-Methyl-1,5,7-triaza-bicyclo[4.3.0]non-6-enium acetate [mTBNH][OAc] to dissolve highly hydrophobic and water-insoluble curcumin. An optimized concentration of 4 mol.kg^-1^ of SBIL was used to dissolve 3.5 mg.g^-1^ of curcumin, resulting in a formulation (curcumin/SBIL) that could be easily dispersed in an aqueous medium. Compared to the aqueous solubility of curcumin alone, the curcumin/SBIL formulation exhibited almost an 8,000-fold increase in solubility, also demonstrating a reduction of ~ 60% of human triple-negative breast cancer epithelial cells (MDA-MB-231) viability with only 10 µg.mL^-1^ of curcumin (the active compound), without any cytotoxic effects on non-tumorogenic mouse fibroblasts (L929). Our study presents a straightforward methodology for improving the solubility and bioavailability of curcumin, which holds promise for its clinical application as an effective anti-cancer drug.

**Supplementary Information:**

The online version contains supplementary material available at 10.1038/s41598-026-44082-7.

## Introduction

**Fig. 1:**
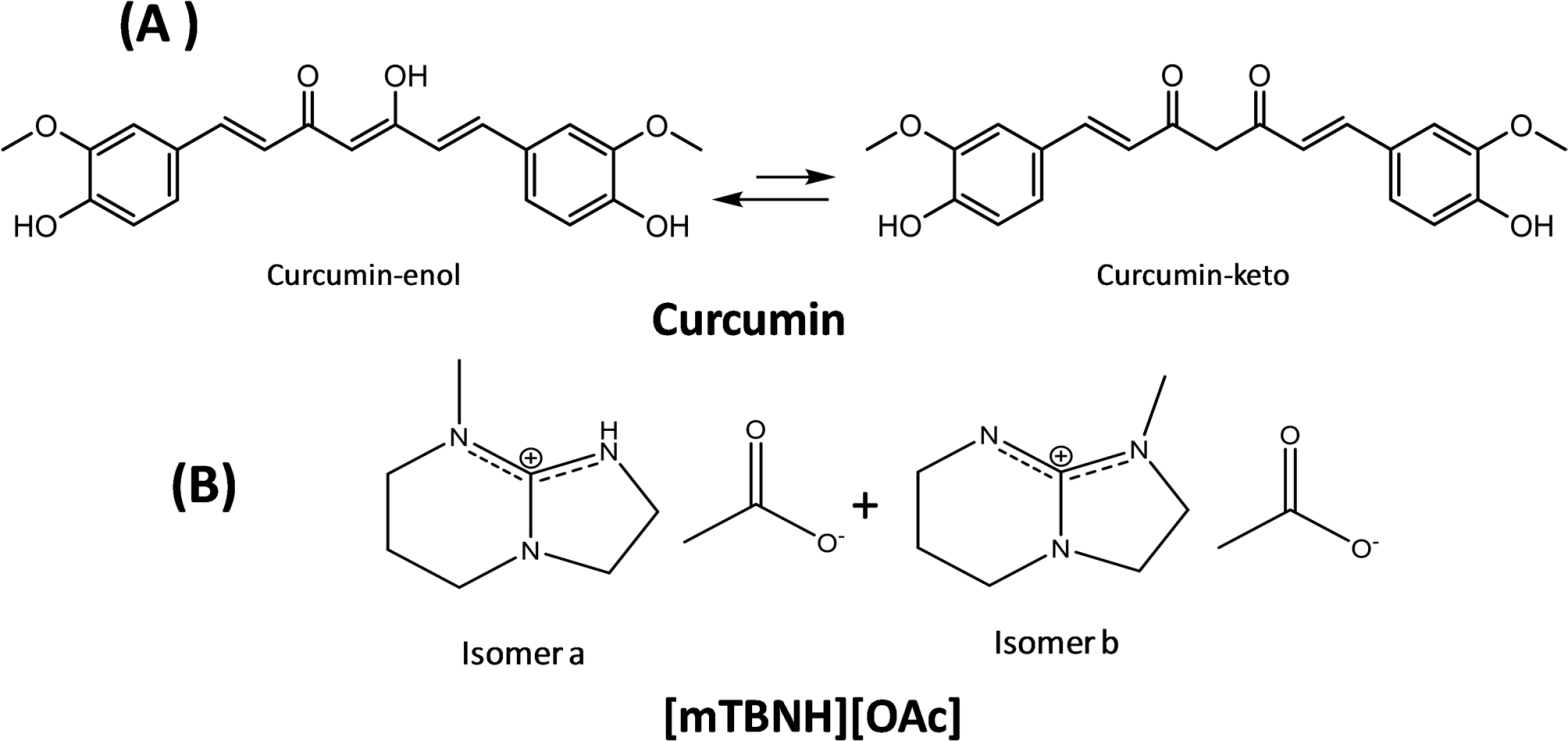
The chemical structure of curcumin and the SBIL [mTBNH][OAc], the latter being a mixture of isomers a and b, which differ in the location of the double bond, and consequently, on the methyl group position.

Curcumin is a diphenolic compound extracted from the rhizome of turmeric, also known as *Curcuma longa*. It is a highly researched phytochemical due to its biological activities, which include anti-inflammatory, antioxidant, antiproliferative, antibacterial, anticancer, and antibiotic properties^1–4^. Curcumin has two significant structural features: phenolic rings and a resonating keto or aldehyde unit (Fig. 1A)^3^. The presence of phenolic rings may disrupt aromatic π − π stacking by interacting with the aromatic residues of structural proteins, while the hydroxyl groups may foster β-sheet breakings and other sugar-based interactions *via* competitive hydrogen bonding^3^. Its potential as an anticancer and Alzheimer’s agent attracted a great deal of research interest^2^. However, the direct application of curcumin is hindered due to its low aqueous solubility and bioavailability^2,3^. Its low bioavailability is due to its rapid hydrolytic degradation under neutral physiological conditions and poor absorption^5,6^.

According to literature, curcumin is known to deeply insert into the cell membrane through hydrophobic interactions with the fatty acid chains, as well as hydrogen bonding with the phosphate groups of the lipids, such as cholesterol. This results in limited diffusion of curcumin into the cytoplasm, which is the main site of action for most of its bioactivity^7^. While it is only soluble in selected organic solvents, such as acetone and methanol, these solvents are unsuitable for biomedical applications, particularly for drug delivery processes, as they are volatile, flammable and toxic^8^. To improve curcumin’s low aqueous solubility and bioavailability, several attempts have been made to encapsulate it inside polymer or lipid-based colloidal particles, such as emulsions^9^, nanoemulsions, micelles^10^, nanoparticles^11^, and liposomes, etc.^5,8,12^. However, these vehicles are inert during drug delivery and the processes involved in their creation can be complex, and both time- and energy-consuming^13^.

Among the various alternative solvents proposed, certain ionic liquids (ILs) have shown potential as biocompatible media that can enhance the dissolution of hydrophobic drugs^14,15^. ILs have been applied in several fields, including drug delivery, synthesis, catalysis, and in the preparation of electrochemical sensors^16,17^. They have been successfully used to solubilize a wide range of poorly soluble compounds, primarily due to their ability to interact with the solute and, if present, co-solvent molecules, which disrupt the existing interactions and promote the solvation of the solute^14,18^. Many studies have focused on developing IL-drug formulations based on various cations such as cholinium, imidazolium, quaternary ammonium, phosphonium and pyrrolidinium^13,19–23^. However, some of the conventional ILs are toxic, chemically unstable, and usually involve multistep synthetic procedures and harsh synthetic conditions^24^. Over the last years, task-specific superbase ILs (SBILs) have been proposed for various applications^25–32^. SBILs have novel properties, including high liquid range, non-volatility, and high thermal and chemical stability. In particular, some common super bases such as 1,1,3,3-tetramethylguanidine (TMG), 1,5,7-triazabicyclo[4.4.0]dec-5-ene (TBD), 1,8-diazabicyclo[5.4.0]undec-7-ene (DBU), 1,5-diazabicyclo[4.3.0]non-5-ene (DBN), have been used to design these ILs^25–30^. Recently, 7-methyl-1,5,7-triazabicyclo[4.4.0]dec-5-enium acetate, [mTBDH][OAc], and 5-methyl-1,5,7-triaza-bicyclo[4.3.0]non-6-enium acetate, [mTBNH][OAc], were identified as promising solvents to achieve cellulose dissolution and fiber regeneration^28,29,32^. These solvents exhibit enhanced thermal stability compared to commonly used structurally similar alternatives^33^. The [mTBNH][OAc] exists as a double-structured IL composed of two isomers, as depicted in Fig. 1B. The mTBN is available in a stoichiometric ratio of 4:3 (a: b) and the resulting IL consists of a mixture of isomers a and b in a certain proportion^30^. In the last five years, these novel SBILs have been applied for dissolution, processing, CO_2_ capture, electrochemistry, spectroscopy, and materials science, but also as additives and catalysts^25,27–32^. However, there are no reports in the field of pharmaceutical drug delivery applications in this regard.

In this context, the objective of this work was to evaluate the solubility of curcumin in aqueous solutions of [mTBNH][OAc], varying in concentration from 0 to 5 mol.kg^− 1^. Subsequently, an optimized concentration (4 mol.kg^− 1^) of SBIL was used to assess the stability of curcumin over time through UV, FTIR, and ^1^H-NMR techniques in the SBIL aqueous solution. Additionally, the degradation kinetics of curcumin in the SBIL aqueous solution were evaluated using degradation models (first-order and second-order models). Finally, the effectiveness of curcumin in the SBIL aqueous solution against human triple-negative breast cancer epithelial cells (MDA-MB-231) and non-tumorogenic mouse fibroblasts (L929) was assessed for cytocompatibility screening assays.

## Materials and methods

### Materials

Curcumin (95% total curcuminoid content) derived from Turmeric rhizome, acetic acid, and dipotassium phosphate - K_2_HPO_4_ (98% of purity) were purchased from Sigma-Aldrich. Potassium persulfate - K_2_S_2_O_8_ (extra pure) was purchased from Scharlau, while Trolox (97%) was purchased from Acros Organic. HPLC-grade methanol (> 99.9% of purity) was obtained from Fisher Scientific and KH_2_PO_4_ (> 99.5%) was purchased from Merck. For cell culture we used GIBCO Dulbecco’s phosphate buffered saline (DPBS), fetal bovine serum (FBS; approved by the EU, and sourced from South America), Dulbecco’s modified Eagle’s medium low glucose (DMEM–low Glc), Dulbecco’s modified Eagle’s medium high glucose (DMEM–high Glc), TrypLETM Express, GIBCO antibiotic/antimycotic solution (ATB) containing 10,000 units.mL^− 1^ of penicillin and 10,000 mg.mL^− 1^ of streptomycin, all of which were purchased from Thermo Fisher Scientific (Alfagene, PT). Adherent 96-well plates from InVitroCell (Sarstedt, PT), non-treated clear bottom 96-well black plates from Corning (Corning, USA) and 13 mm round-treated coverslips from Sarstedt (Sarstedt, PT) were used. Calcein-AM, Propidium Iodide (PI), DAPI and Fluorescein Phalloidin were all purchased from Thermo Fisher Scientific Inc (Alfagene, PT). The 5-Methyl-1,5,7-triaza-bicyclo[4.3.0]non-6-enium acetate ([mTBNH][OAc], purity > 97%)] was kindly supplied by the University of Helsinki, prepared according to the procedure detailed in a previous study^30,34^. The water content of the compounds was determined using a Metrohm 831 Karl Fischer coulometer with Hydranal^®^–Coulomat AG from Riedel-de Haën as the analyte. All samples were prepared using distilled water.

### Methods

#### Solubility and stability of the curcumin/SBIL formulation

To determine the solubility of curcumin, excess amounts of curcumin were added to microcentrifuge tubes containing various concentrations of SBIL, which were then mixed using a vortex for 1 h and allowed to reach equilibrium under ambient conditions. The samples were subsequently centrifuged at 13,000 rpm for 10 min to remove any undissolved parts, as previously reported^35^. The stability of curcumin (3.5 mg·g^-1^) in SBIL (4 mol·kg⁻¹) was investigated, as this concentration exhibited the highest curcumin solubility among those tested. Samples were prepared in triplicate, kept in sealed tubes protected from light and stored in a temperature-controlled oven at 25.0 ± 0.5 °C. Aliquots were withdrawn on days 0, 1, 3, 5, 7, 10, 14, and 21 for analysis.

UV, FTIR, and NMR analyses were employed. For UV absorbance profiling, 10 µL of SBIL containing curcumin was added to 990 µL of MilliQ water, thoroughly mixed, and the profile measured. For FTIR analysis, a PerkinElmer Spectrum BX spectrometer equipped with a single horizontal Golden Gate ATR cell was used. The spectra were obtained with 32 scans at a resolution of 2 cm^− 1^ and a wavenumber range between 4000 and 600 cm^− 1^. For ^1^H-NMR analysis, the samples were dissolved in deuterated dimethyl sulfoxide, and all spectra were recorded using a Bruker Avance 300 NMR spectrometer at 300 MHz. The time-dependent stability of curcumin within SBIL was established by storing the formulation in dark at room temperature for 3 weeks. The degradation data were fitted to first- and second-order kinetic models using Microsoft Excel. Eight time points (from the triplicate samples) were used for the fitting^36^. The Solver tool in Excel was employed for regression analysis, and the best-fitting kinetic model was selected based on the highest coefficient of determination (R²).

#### Cell culture conditions

Human triple-negative breast cancer epithelial cells (MDA-MB-231), non-tumorigenic fibroblasts (L929), both from ATCC, were cultured in DMEM high glucose and DMEM low glucose, respectively, supplemented with 10% FBS, 100 U.mL^− 1^ of penicillin, and 0.1 mg.mL^− 1^ of streptomycin (Thermo Fisher Scientific). The cellular cultures were maintained in a humidified incubator at 37 °C with 5% of CO_2_.

#### Preparation of SBIL and SBIL/curcumin stock solutions for cell viability tests

To prepare the SBIL and SBIL/curcumin stock solutions for the cell viability tests, we diluted fresh SBIL and curcumin samples in the cell culture media (DMEM high or low glucose) at a ratio of 1:4 (v/v). Stock solutions of curcumin/SBIL were prepared at a concentration of 1 mg.mL^− 1 ^(where the concentration of SBIL was 4 mol.kg^-1^), sterilized by filtration, and stored at 4 °C until use.

#### Cell viability

To begin the experiment, cells (MDA-MB-231 and L929) were seeded at a density of 1,000 cells *per* well on 96-well plates or 5,000 cells *per* well on an 8-well IBIDI µ-slide and allowed to adhere overnight. After adherence, the culture media was replaced by SBIL/curcumin previously diluted in the designated culture media to achieve a curcumin concentration of up to 10 µg.mL^− 1^. After 24 h, the metabolic activity of the cells was determined using the AlamarBlue assay (Thermo Fisher Scientific, USA), according to the manufacturer’s instructions. Briefly, 10% of AlamarBlue buffer was added to each well and incubated for 4 h. Afterward, the media were transferred to a 96-well black plate with clear bottom, and the fluorescence intensity was measured using a multimode microplate reader. To assess the cell viability, a Live/Dead assay was also performed after 24 h incubation. MDA-MB-231 and L929 cells were washed with 1x Dulbecco’s phosphate-buffered saline (DPBS) and then incubated with Calcein-AM (3 µg.mL^− 1^) and Propidium Iodide (PI, 6 µg.mL^− 1^) for 30 min at 37 °C. The cells were then rinsed with DPBS and immediately observed using LSM fluorescent microscopy.

#### Statistical analysis

All statistical analyses were performed by GraphPad (Prism 9.00), using two-way ANOVA with Tukey’s post-test; statistically significant considered for *p-value < 0.05. Four independent trials were performed, and the data are expressed as mean ± standard error (SE).

## Results and discussion

### Solubility and stability of the curcumin/SBIL formulation

**Fig. 2:**
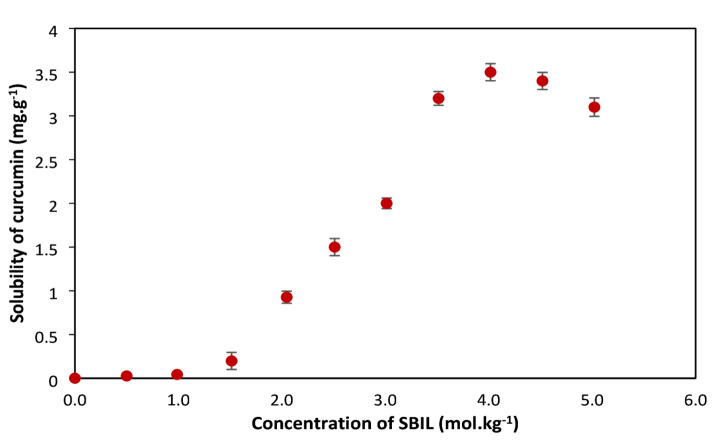
Solubility (mg.g^− 1^) of curcumin in various concentrations of SBIL [mTBNH][OAc].

To understand the influence of SBIL concentration on the dissolution of curcumin, we conducted an initial assessment of the solubility data of curcumin in aqueous solutions containing different amounts of [mTBNH][OAc] at a temperature of 298.15 K, as depicted in Fig. 2. Based on the solubility data, curcumin’s water solubility is extremely low, with a value of approximately 0.4 × 10^− 3^ mg·g^−1^ at pH 7.3 (distilled water). Due to the extremely low water solubility of curcumin, no UV absorption was detected in pure water, whereas a characteristic UV peak of curcumin was observed at approximately 426 nm for the curcumin/SBIL formulation (Figure S1, A). For clarity, the background interference of SBIL in aqueous solution is also provided in Figure S1, B.

The solubility of curcumin tends to increase with the rise in molar concentration of SBIL in the aqueous solution, reaching a maximum solubility of 3.5 mg·g^−1^ at a concentration of 4.0 mol·kg^−1^ of SBIL, representing an 8750-fold increase compared to its solubility in pure water. For comparison with other IL-based systems, previous studies have investigated the effect of alkyl chain length for imidazole ILs such as [C_6_MIM]Br, [C_8_MIM]Br, [C_10_MIM]Br, [C_12_MIM]Br, as well as quaternary ammonium ILs ([N_2,2,2,8_]Br, [N_2,2,2,10_]Br, [N_2,2,2,12_]Br, and [N_2,2,2,14_]Br)^37^. At concentrations up to 300 mM of IL, these studies reported curcumin solubility of approximately 4 mg·mL⁻¹, which is of the same order of magnitude as observed in our work. However, the IL proposed in this study offers advantages in terms of lower toxicity and reduced cost. This strong solubilization effect can be attributed to hydrotropic interactions between the SBIL ions and curcumin molecules. Specifically, the amphiphilic nature of the SBIL promotes π–π stacking and hydrogen-bonding interactions with the aromatic and polar groups of curcumin, while simultaneously reducing the interfacial tension between curcumin and water. At higher concentrations (> 4.0 mol·kg⁻¹), SBIL aggregation likely limits the availability of free ions capable of interacting with curcumin, leading to a slight decrease in solubility. The resulting solubility profile, with a maximum at intermediate SBIL concentrations, is characteristic of hydrotropic systems, and similar behavior has been observed for other amphiphilic solutes in SBIL aqueous solution^38–40^.

To investigate curcumin stability in the SBIL aqueous solution (4 mol·kg^−1^), UV-Vis, FTIR, and ^1^H-NMR techniques were utilized. The infrared spectra of curcumin solubilized in SBIL aqueous solution (Figure S2) display a typical [mTBNH][OAc] pattern, with fingerprints in the regions 1342–1266 cm^−1^ and 1250 − 1020 cm^−1^ related to the stretching vibration of aromatic amines (C-N stretching) and the curcumin signal at 1628 cm^−1^ related to the stretching vibrations of carbonyl (C = O) and alkenes (C = C). A strong band at 1512 cm^−1^ indicates mixed vibrations involving aliphatic, aromatic, keto, and enol configurations (including ν C = O, δ CC-C, δ CC = O, δ CC-H, and ν CC). Additionally, a pronounced band at 1277 cm^−1^ corresponding to the phenolic band (ν C-O) was observed^41–43^. All observed bands refer to either SBIL or curcumin at the given concentrations, and no new bands were observed, indicating that no degradation of curcumin occurred.

To further evaluate this, ^1^H-NMR spectra of both SBIL (4 mol.kg^−1^) and curcumin (3.5 mg.g^−1^) solubilized in SBIL aqueous solution were obtained. Figure S3 shows the chemical shift spectra of the SBIL aqueous solution before and after curcumin solubilization. There was no significant difference in chemical shift between the signals of pure and solubilized curcumin in SBIL, indicating that the dissolved curcumin remained stable without modifications to its chemical structure. These results suggest that there are no significant changes in the structure of curcumin when solubilized in the SBIL aqueous solution. However, time is another crucial factor. The time-dependent stability, or shelf-life, of curcumin is essential for preserving its pharmaceutical activity. Therefore, it was our objective to check the stability of 4 mol.kg^−1^ of SBIL in water after long-term storage. The ^1^H-NMR spectra of the stored SBIL aqueous solution showed no structural changes in SBIL, even after 3 weeks (Figure S4). However, a new peak (~ 3.9 ppm) corresponding to water was observed (Figure S4). The chemical shift of hydrogens in the acetate of [mTBDH][OAc] remained consistent at 1.60 ppm for both fresh and stored samples (Figure S4).

**Fig. 3:**
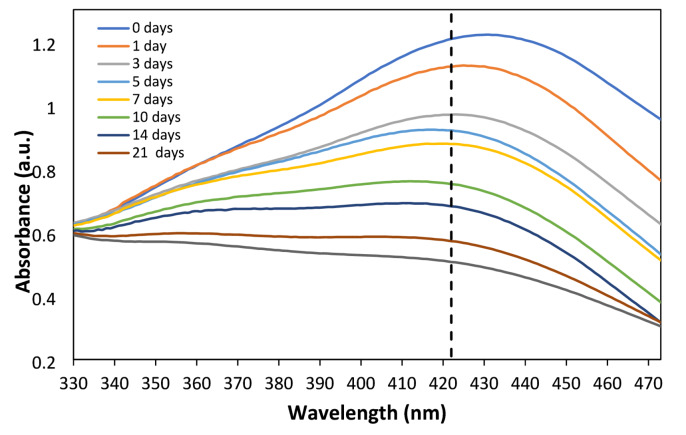
UV-Vis absorbance spectrum of curcumin after storing the curcumin/SBIL formulation at room temperature for 4 weeks (28 days).

To better understand the degradation behavior of curcumin, UV spectra of SBIL aqueous solution with dissolved curcumin were obtained over 28 days (Fig. 3). Based on the UV spectra, there is an almost linear decrease in the curcumin peak (422 nm) over time. Furthermore, no shift in the maximum absorbance peak of curcumin was detected for up to 21 days, implying that the remaining curcumin has retained its original molecular structure.

Kinetic data were fitted using first-order and second-order degradation models (Table 1). The data presented in Table 1 and Figure S5 show a better fit to the second-order model (R² = 0.994 vs. 0.970 for first-order), indicating a mechanistic shift induced by the SBIL environment. In aqueous medium, curcumin degradation typically follows first-order kinetics, where the rate is proportional to the curcumin concentration, and is influenced by factors such as pH, temperature, and the dielectric constant of the medium^44^. In the presence of SBIL, curcumin can form complexes or aggregates with SBIL molecules, which likely promotes bimolecular interactions and leads to the observed second-order degradation kinetics. These interactions may involve catalytic species, altered solvation, or microenvironmental effects that favor intermolecular reactions such as self-aggregation, nucleophilic attack, or redox processes. Additionally, the self-buffering nature of SBIL may stabilize curcumin, highlighting the critical role of SBIL’s chemical architecture in modulating curcumin stability and reactivity. The observed half-life of curcumin in the SBIL aqueous solution was approximately 385 h, about 38 times longer than the half-life of curcumin in aqueous solution (8–10 h)^45,46^. Our measured degradation rate constant (k = 0.0018 h⁻¹ = 0.043 day⁻¹) is significantly lower than values reported for most common solvents, including water (k ≈ 0.31 day⁻¹), ethanol, DMSO, and surfactants, and comparable to some ILs such as [C_4_mim][C₈SO₄]^47^. This confirms that our SBIL provides exceptional stabilization against curcumin degradation, likely due to its unique microenvironment that suppresses hydrolysis and oxidation. While a few ILs show marginally slower kinetics, our system offers a compelling combination of high stability, biocompatibility, and formulation versatility. Moreover, our measured degradation rate constant (k = 0.0018 h⁻¹, or ~ 0.043 day⁻¹) is substantially lower than values reported in literature for common organic solvents and other ILs, such as DMSO (0.002), ethanol, [C_4_mim][BF₄], and [C_4_mim][C₈SO₄], which typically exhibit k values an order of magnitude higher^48^. This underscores the exceptional stabilizing capacity of our SBIL, likely due to its tailored microenvironment that effectively suppresses curcumin degradation pathways such as hydrolysis and oxidation.

**Table 1:** Kinetic parameters were obtained using first-order kinetics and a second-order model to assess the influence of SBIL aqueous solutions on the stability of curcumin at 298.15 K.

Kinetic models	k_d_ (h^− 1^)	t_1/2_ (h)	*r* ^2^
First-order	0.0015	462.1 (~ 19 days)	0.970
Second-order	0.0018	385.1 (~ 16 days)	0.994

### Cytocompatibility of the curcumin/SBIL formulation

**Fig. 4:**
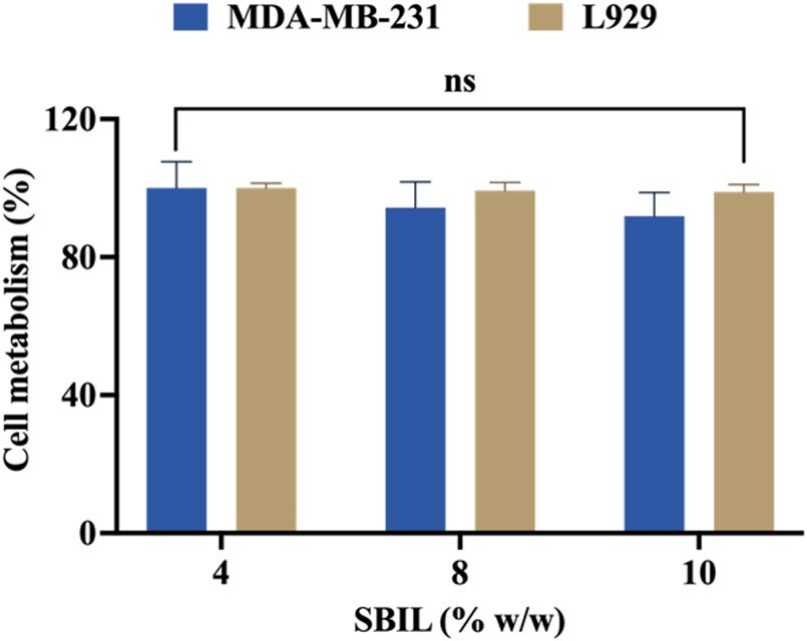
Evaluation of the SBIL effect on the cell metabolism of human triple-negative breast cancer epithelial cells (MDA-MB-231), non-tumorigenic mouse fibroblasts (L929). Statistical significance was set at *p* < 0.05: ns = non-significant; *n* = 3 replicates.

To evaluate the effectiveness of curcumin in the aqueous solution of SBIL against human triple-negative breast cancer epithelial cells (MDA-MB-231), non-tumorigenic mouse fibroblasts (L929) were chosen as healthy cells for cytocompatibility screening assays. Different concentrations of SBIL were tested on both L929 and MDA-MB-231 cells to evaluate SBIL cytotoxicity and to optimize its concentration for further studies (Fig. 4). The results showed good cytocompatibility for concentrations up to 10 wt% (0.5 mol.kg^−1^) of SBIL for both cell types, indicating no toxic effects from SBIL alone. This means that with this concentration of SBIL (0.5 mol·kg^−1^), it is possible to solubilize approximately 65 times more curcumin in water without any toxic effects on the cells.

**Fig. 5:**
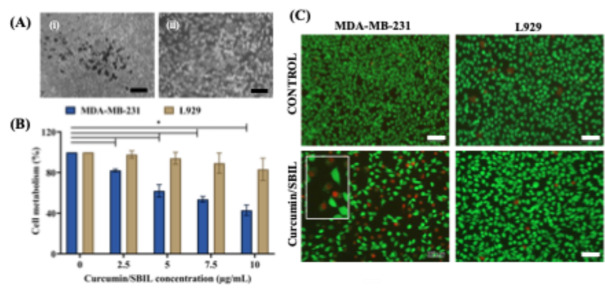
Evaluation of curcumin/SBIL formulations in human triple-negative breast cancer epithelial cells (MDA-MB-231) and mouse fibroblasts (L929). (**A**) Light microscopy of L929 cells incubated with (i) curcumin powder and (ii) curcumin/SBIL formulation in DMEM low glucose. Scale bars represent 100 μm. (**B**) Cell metabolism of cancer cells and fibroblasts exposed to several curcumin/SBIL concentrations. Statistical significance was set at *p* < 0.05: *p < 0.05, *n* = 3 replicates. (**C**). Representative live/dead images of MDA-MB-231 and L929 incubated with curcumin/SBIL (10 µg.mL^− 1^) for 24 h. Live cells are stained by calcein-AM (green), and dead cells with propidium iodide (red). Scale bars represent 100 μm.

In a second test, the curcumin was initially dissolved in water and in the optimized SBIL aqueous solution and mixed with cell culture media before added to the cells. We tested different concentrations of SBIL, ensuring cytocompatibility by keeping the SBIL amount below 10 wt% (0.5 mol kg^−1^). The curcumin aqueous solution showed precipitation behavior, and dark aggregates were formed, invalidating homogeneous exposure to cells (Fig. 5A-i). Under these biologically relevant conditions, curcumin is known to rapidly aggregate due to its extremely low intrinsic aqueous solubility, limiting its bioavailability^49^. The curcumin/SBIL formulation dissolve perfectly in the cell culture media without any evident aggregation behavior (Fig. 5A-ii). This resulted in noticeable differences in cell metabolism between cancer and healthy cells, as shown in Fig. 5B. Specifically, MDA-MB-231 cancer cells exhibited a significant reduction in metabolism (~ 60%) when treated with 10 µg.mL^−1^ of curcumin/aqueous solution of SBIL, compared to the control. In contrast, non-tumorogenic L929 cells did not show a significant reduction under the same conditions. Although we were not able to reach the IC_50_ at the single 24‑hour time point, we observed approximately a 60% reduction in cell viability at 10 µg.mL^−1^ (~ 27 µM). In contrast, in many curcumin studies for the same cell line, IC₅₀ values are typically observed at longer exposure durations (48–72 h)^50,51^. This indicates that our formulation accelerates curcumin uptake and cytotoxic activity in these cells. We further confirmed the effectiveness of the curcumin/SBIL formulation through live/dead assay, which revealed that the highest concentration of the formulation disrupted the membrane integrity of the cancer cells, indicative of late necrosis/apoptosis (Fig. 5C). Furthermore, the curcumin/SBIL formulation was found to reduce the density of cancer cells after 24 h of incubation, highlighting the potent cytotoxic effect of the formulation. Importantly, this reduction did not negatively affect the membrane integrity of L929 cells, as evidenced by the lower number of dead cells and the higher cell density observed.

The tested SBIL concentrations did not exhibit cytotoxicity, suggesting that curcumin is the primary contributor to the cancer cell death observed. Several studies have confirmed the anti-cancer properties of curcumin, including the inhibition of tumor proliferation, evasion/metastasis, and other effects^52,53^. Moreover, curcumin has demonstrated promising results in breast cancer treatment, with multiple studies, indicating its modulation of the tumor microenvironment through various cellular signaling pathways^53–56^. However, using curcumin in clinical practice is hindered by its poor aqueous solubility and low bioavailability^57,58^ leading to the development of different types of nanoparticle-based systems for improvement of delivery pathways and early stage detection of cancer^55^^[,59^^[,60^, These approaches can be complicated and time-consuming. Our approach offers a simple solution to enhancing curcumin’s bioavailability and anti-cancer efficiency while preserving healthy cells.

## Conclusions

Nanocarrier-based curcumin formulations have emerged as essential strategies to overcome the major limitations of native curcumin, mainly its extremely low aqueous solubility and poor bioavailability. The present work shows that SBIL-based formulations can significantly enhance the solubility of curcumin and hence its therapeutic efficacy. This study evaluated the solubilization and stabilization capacity of SBIL aqueous solution for curcumin. The SBIL-based formulation showed improved solubility, stability, and in vitro anticancer activity, making it a promising approach for developing tumor-targeted therapeutics. We were able to dissolve ~ 3.6 mg.g^− 1^ of curcumin in this formulation without compromising its structure, and its stability, which was confirmed through ^1^H-NMR and FTIR spectra after long-term storage. The lack of cytotoxicity of the [mTBNH][OAc] and improved solubility of curcumin enabled us to reduce the viability of breast cancer cells by ~ 60% after just 24 h of contact with 10 µg.mL^− 1^ of curcumin/SBIL aqueous solution. This approach can be used to dissolve various hydrophobic drugs and enhance their anti-tumor efficiency, expanding the scope of SBIL in pharmaceutical sciences.

## Supplementary Information

Below is the link to the electronic supplementary material.

Supplementary Material 1

## Data Availability

All data supporting the findings of this study are available within the paper and its Supplementary Information.
