# Supplementary material for: Superbase ionic liquid mediated solubilization of curcumin for improved bioavailability and anticancer efficacy
Source: Sci Rep. 2026 Apr 17;16:17923. doi: 10.1038/s41598-026-44082-7 (PMC13249852; doi:10.1038/s41598-026-44082-7)
Supplement: Supplementary file 1 — Supplementary Material 1 [file 41598_2026_44082_MOESM1_ESM.docx]

**Superbase ionic liquid mediated solubilization of curcumin for improved bioavailability and anticancer efficacy**

Meena Bisht^a#*^, Maria C. Gomes^b#^, Filipe Hobi Bordon Sosa^b^, João F. Mano^b^, Siddharth Pandey^c^, Sónia P. M. Ventura^b*^, João A. P. Coutinho^b^

^a^Department of Chemistry, Sri Venkateswara College, University of Delhi, Dhaula Kuan, New Delhi 110021, India.

^b^Department of Chemistry, CICECO-Aveiro Institute of Materials, University of Aveiro, 3810-193, Aveiro, Portugal.

^c^Department of Chemistry, Indian Institute of Technology Delhi, Hauz Khas, New Delhi –110016, India.

# MB, MCG, and FHBS contributed equally

*Corresponding authors:

Emails:

Sónia P. M. Ventura: [spventura@ua.pt](mailto:spventura@ua.pt)

Meena Bisht: [meena@svc.ac.in](mailto:meena@svc.ac.in)


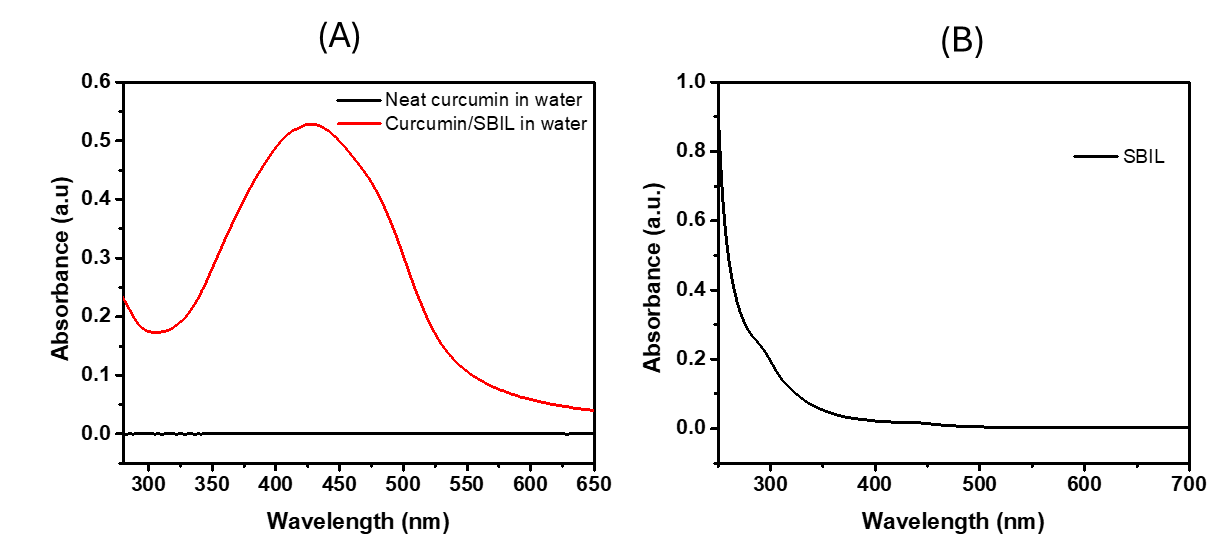


**Figure S1.** (A) UV spectra of pure curcumin and curcumin/SBIL formulation in aqueous solution and (B) background interference of SBIL in aqueous solution.

**Figure S2.** FTIR−ATR spectra of curcumin and SBIL aqueous solution (80 wt%) before and after curcumin solubilization.


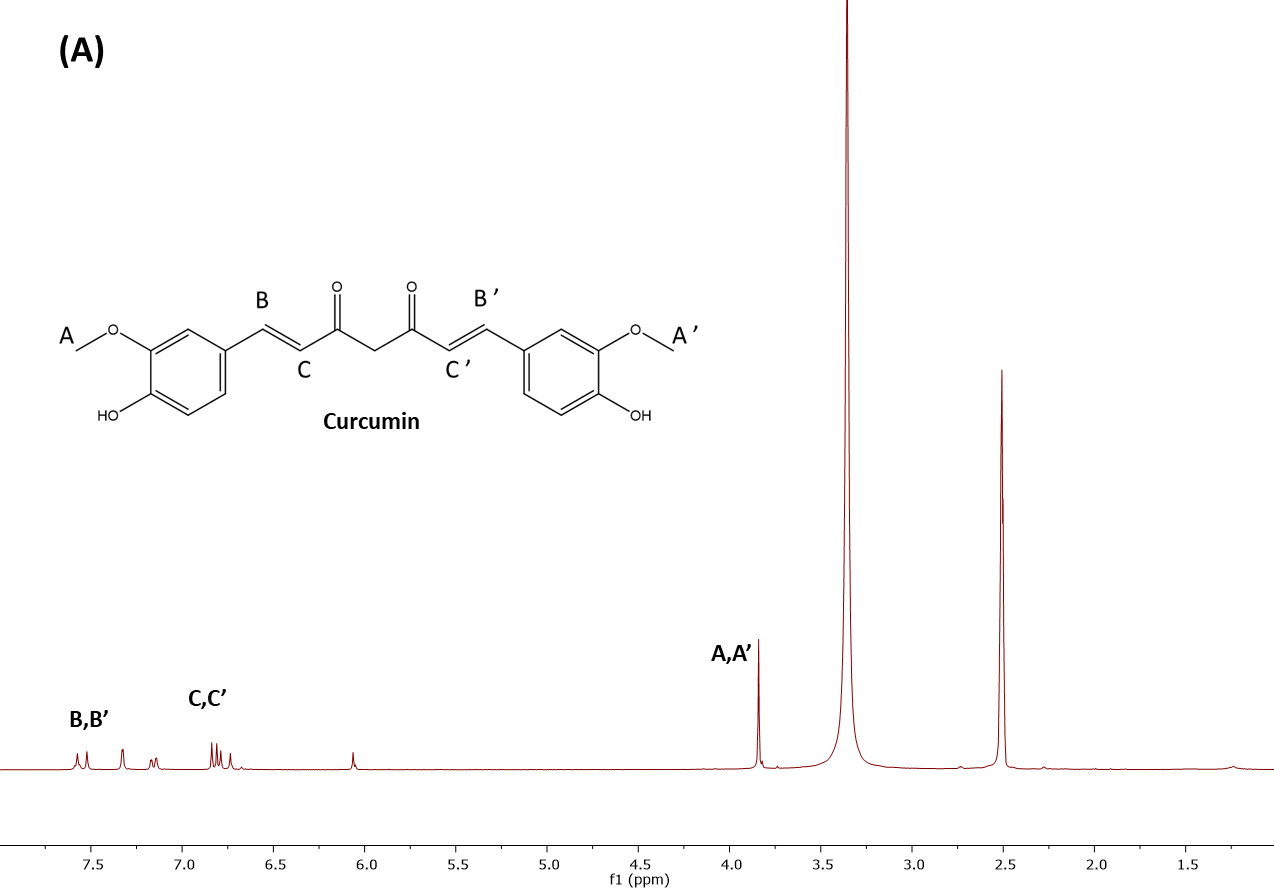


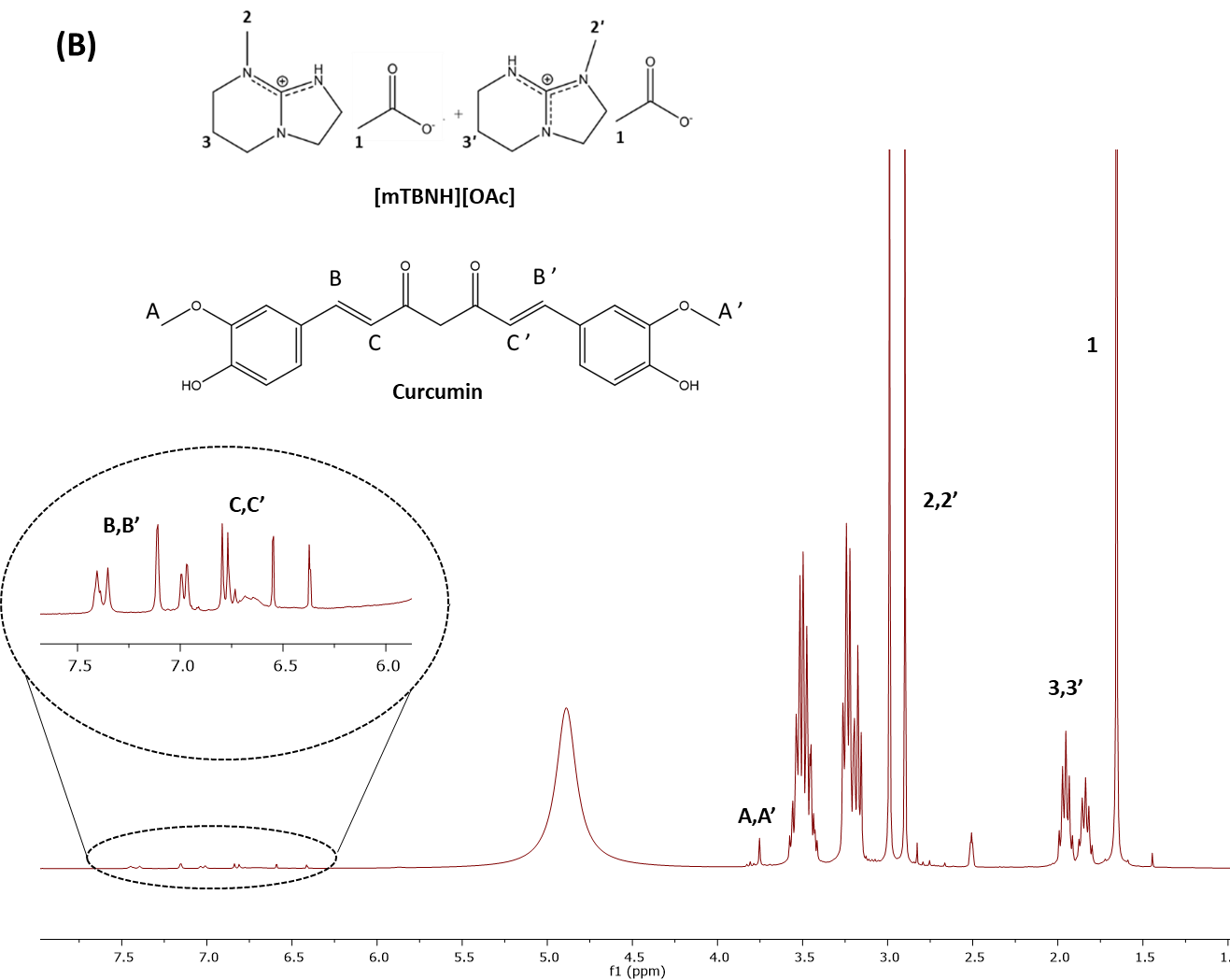


**Figure S3.** ^1^H-NMR spectra of (A) curcumin and (B) SBIL 80 wt% with dissolved curcumin.

**
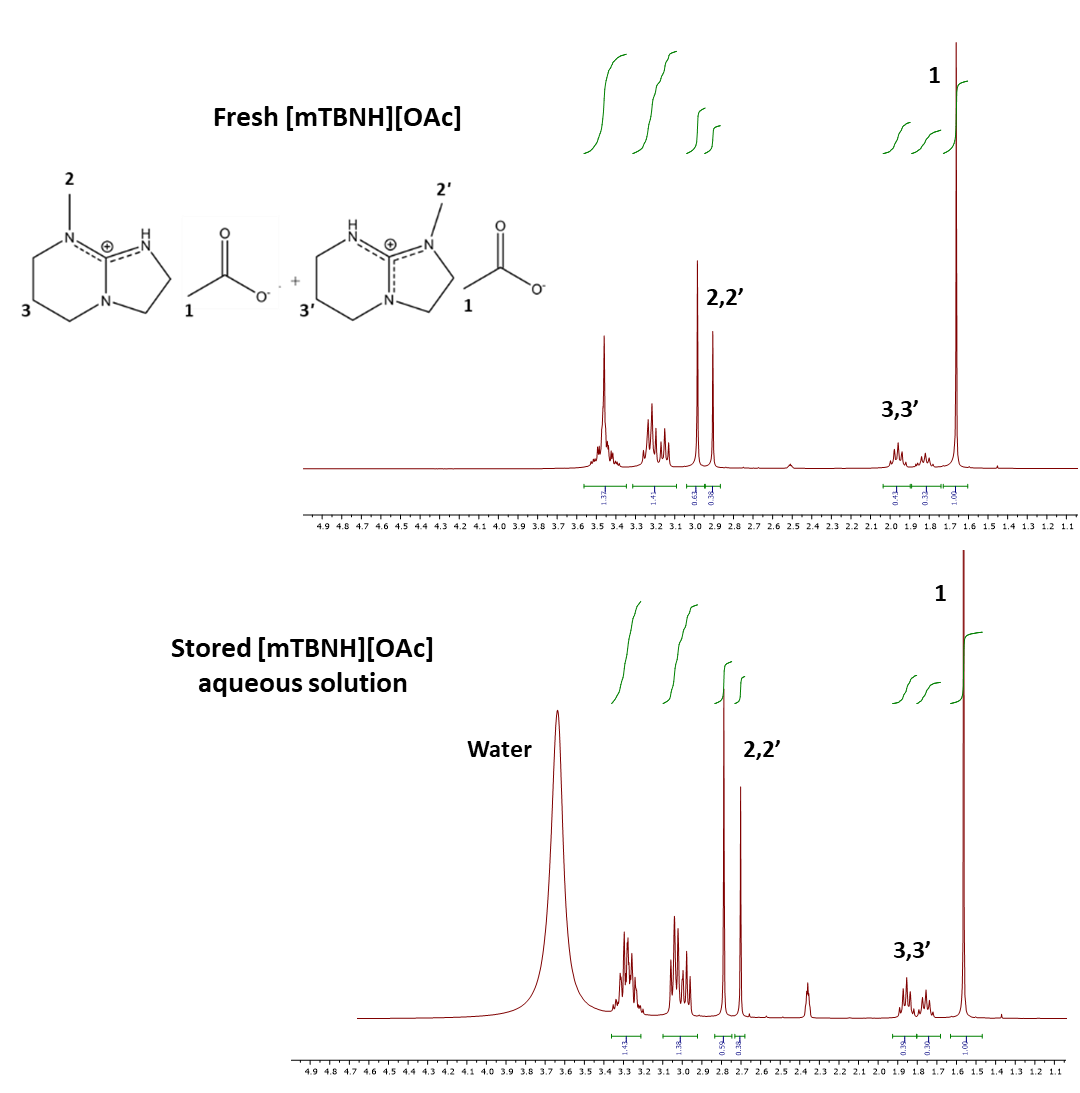
**

**Figure S4.** ^1^H**-**NMR spectra of fresh SBIL and aqueous solution of SBIL 80 wt%, after 3 weeks.

**
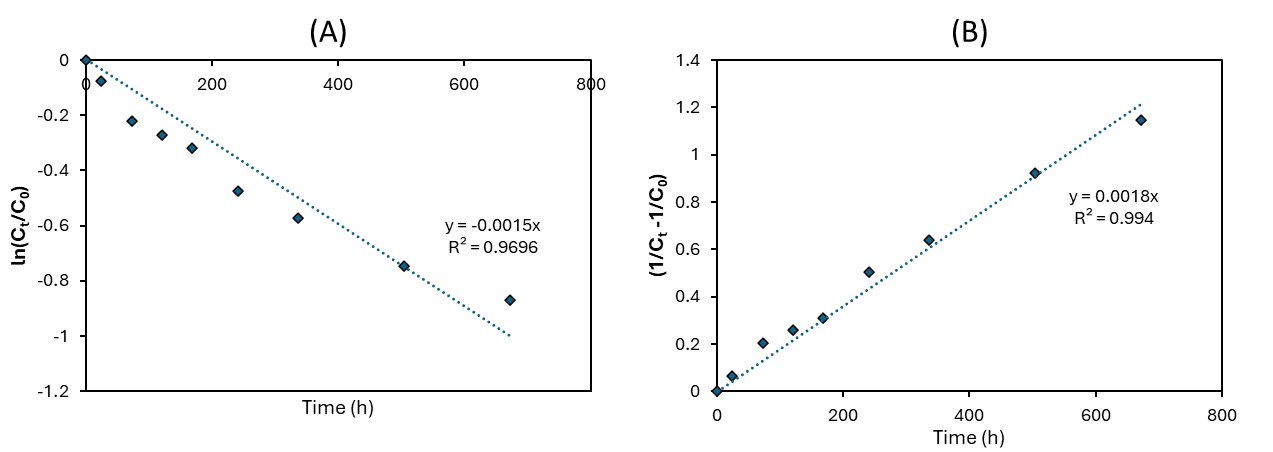
**

**Figure S5.** Kinetics of curcumin’ degradation considering (A) first-order and (B) second-order models.
